# Gut fungi of black-necked cranes (*Grus nigricollis*) respond to dietary changes during wintering

**DOI:** 10.1186/s12866-024-03396-0

**Published:** 2024-06-29

**Authors:** Wenhao Li, Lijun Cheng, Xin He, Guiwen He, Yutong Liu, Zhenglin Sang, Yuanjian Wang, Mingcui Shao, Tingsong Xiong, Huailiang Xu, Junsong Zhao

**Affiliations:** 1 College of Agronomy and Life Sciences, Zhaotong University, Zhaotong, 657000 China; 2 Yunnan Key Laboratory of Gastrodia and Fungi Symbiotic Biology, Zhaotong University, Zhaotong, 657000 China; 3 College of Life Science, Sichuan Agricultural University, No. 46, Xinkang Road, Yucheng District, Ya’an, Sichuan 625014 China; 4 Sichuan Academy of Grassland Sciences, Chengdu, 610000 China; 5 Management Bureau of Dashanbao Black-Necked Crane National Nature Reserve, Zhaotong, Yunnan Province 657000 China

**Keywords:** Black-necked crane, Diet, Gut fungi, Wintering period

## Abstract

**Background:**

Migratory birds exhibit heterogeneity in foraging strategies during wintering to cope with environmental and migratory pressures, and gut bacteria respond to changes in host diet. However, less is known about the dynamics of diet and gut fungi during the wintering period in black-necked cranes (*Grus nigricollis*).

**Results:**

In this work, we performed amplicon sequencing of the trnL-P6 loop and ITS1 regions to characterize the dietary composition and gut fungal composition of black-necked cranes during wintering. Results indicated that during the wintering period, the plant-based diet of black-necked cranes mainly consisted of families Poaceae, Solanaceae, and Polygonaceae. Among them, the abundance of Solanaceae, Polygonaceae, Fabaceae, and Caryophyllaceae was significantly higher in the late wintering period, which also led to a more even consumption of various food types by black-necked cranes during this period. The diversity of gut fungal communities and the abundance of core fungi were more conserved during the wintering period, primarily dominated by Ascomycota and Basidiomycota. LEfSe analysis (*P* < 0.05, LDA > 2) found that *Pyxidiophora*, *Pseudopeziza*, *Sporormiella*, *Geotrichum*, and *Papiliotrema* were significantly enriched in early winter, *Ramularia* and *Dendryphion* were significantly enriched in mid-winter, *Barnettozyma* was significantly abundant in late winter, and *Pleuroascus* was significantly abundant in late winter. Finally, mantel test revealed a significant correlation between winter diet and gut fungal.

**Conclusions:**

This study revealed the dynamic changes in the food composition and gut fungal community of black-necked cranes during wintering in Dashanbao. In the late wintering period, their response to environmental and migratory pressures was to broaden their diet, increase the intake of non-preferred foods, and promote a more balanced consumption ratio of various foods. Balanced food composition played an important role in stabilizing the structure of the gut fungal community. While gut fungal effectively enhanced the host’s food utilization rate, they may also faced potential risks of introducing pathogenic fungi. Additionally, we recongnized the limitations of fecal testing in studying the composition of animal gut fungal, as it cannot effectively distinguished between fungal taxa from food or soil inadvertently ingested and intestines. Future research on functions such as cultivation and metagenomics may further elucidate the role of fungi in the gut ecosystem.

**Supplementary Information:**

The online version contains supplementary material available at 10.1186/s12866-024-03396-0.

## Introduction

The gut microbiota composition is directly linked to the environmental adaptability of avian hosts [1–3]. The gut microbiota modulates the adaptability of wild birds further through immune responses, reproductive fitness, physiological reactions, reproductive behaviors, and cognition [3–6]. Meanwhile, the gut microbiota of birds is influenced by various factors such as genetics [7, 8], diet [9, 10], and environment [11, 12]. Among these, diet has been found to play a significant role in shaping avian microbiota [10, 13, 14]. Studies on the gut microbiota composition of Darwin’s finches found that the effect of diet surpasses phylogenetic relationships [8].

The difficulty in assessing dietary components under uncontrolled conditions has led to fewer studies on the composition of wild bird food structures. However, with the advancement of high-throughput sequencing technologies, DNA barcoding has emerged as a powerful tool for assessing wild animal diets, with primer sets for amplifying rbcL and trnL (UAA) being widely used for revealing the composition of plant-based food structures [15–18]. For migratory birds, habitat plays a crucial role in their survival [19]. Suitable winter climate and abundant food resources in wintering areas ensure the survival of migratory birds during the winter [20, 21]. Driven by food availability, flexible habitat selection and foraging strategy changes are the primary behavioral adaptations of migratory birds to winter environments [22–24]. Migratory birds increase foraging (i.e., hyperphagia) in the late wintering period, leading to fat accumulation and energy reserves for spring migration [25–29]. Although some understanding of the diet composition of the eastern population of black-necked cranes during winter has been gained through fecal examination [30] and behavioral monitoring [31], knowledge of the dynamics of their winter diet composition remains limited.

The host’s feeding behavior is an important pathway for the exchange of microbes both internally and externally [32]. By controlling the gut microbiota to adapt to changes in food supply, the host plays a crucial role in maintaining animal nutrition absorption and health [9, 33]. As a member of the gut microbiota, fungi have been shown to be closely associated with the dietary digestion [34, 35] and immune regulation [36] of the host. Among the fungi with the highest abundance in the gut of cranes, the phylum Ascomycota has been identified as the major cellulose-degrading fungi [37, 38], which can secrete large quantities of cellulases and hemicellulases to facilitate the breakdown of complex polysaccharides in the food of the host [39, 40], thus improving the digestibility of the food in the host [38, 41–43]. Aspergillus are abundant in the gut fungal of cranes, and some plant saprophytic fungi are also believed to originate from food sources [44], which fully illustrates the significant impact of food on the composition of bird gut fungal. However, the relationship between gut fungal communities and diet in birds still requires further investigation. Meanwhile, current research on how the gut microbiota influences the disease status of avian hosts is relatively limited, but Noguera et al. (2018) suggest that the risks associated with stress exposure and pathogen infection in wild birds are closely related to the gut microbiome [45]. The pathogens in the gut fungal are also believed to pose a potential threat to the ecological health of the host [46]. The pathogens in the gut fungal are also believed to pose a potential threat to the ecological health of the host and these pathogenic fungal taxa could be acquired from the diet. Hence why it is interesting to look at the correlation between diet and gut fungal communities.

The black-necked crane (*Grus nigricollis*) is a globally vulnerable species and is the only crane that spends its entire life at high altitudes [19]. It also is known as an environmental indicator and flagship species in alpine wetland ecosystem [47]. They are mainly found on the Qinghai-Tibet and Yunnan-Guizhou Plateaus in China, with the eastern population in the Dashanbao Wetland Nature Reserve migrating to the Ruoergai Grassland for breeding and summering from late March to early April each year, and returning for wintering in December [48, 49]. Our monitoring data from the winter of 2022 showed that more than 1,700 individuals were stably inhabiting the Dashanbao, making it ideal for the study of this treasured species. Through behavioral monitoring, it was found that during the stable period of winter, the diet of black-necked cranes in the Caohai National Nature Reserve in Guizhou is mainly composed of plant-based foods, supplemented by a small amount of animal-based foods [50]. Monitoring of foraging behavior of black-necked cranes in Dashanbao also showed that the consumption of potatoes is significantly higher in the early wintering period, while the consumption of arthropods is higher in the later wintering period [31]. During sampling, we also noticed that black-necked cranes concentrate on consuming potatoes in potato fields in the early wintering period. Meanwhile, local residents also feed small amounts of corn to black-necked cranes at fixed locations throughout the winter to supplement their food. Previously, we systematically revealed the gut bacteria of black-necked cranes during the wintering period in Dashanbao by 16 S rDNA amplicon and metagenomic approaches [51, 52]. We found that the gut microbiota adapted to changes in the host diet during the wintering period and adjusted its function by altering the abundance of core microbial populations, which may help black-necked cranes accumulate energy before migration [52]. However, less is known about the relatively small number of fungal members in the gut. This study characterized the dynamics of plant food composition and gut fungal communityin the Dashanbao area during the pre-wintering, mid-wintering, and late-wintering periods of black-necked cranes using amplicon sequencing and DNA barcoding technology. The aim was to reveal how black-necked cranes adjust their foraging strategies during the wintering period to better adapt to their environment. Furthermore, the study analyzed the response of black-necked crane winter gut fungal communities to changes in their diet, and explored the potential relationship between the gut fungal composition structure of black-necked cranes and their feeding behavior, providing a theoretical basis for the effective protection of black-necked cranes.

## Materials and methods

### Study area and sample collection

**Fig. 1:**
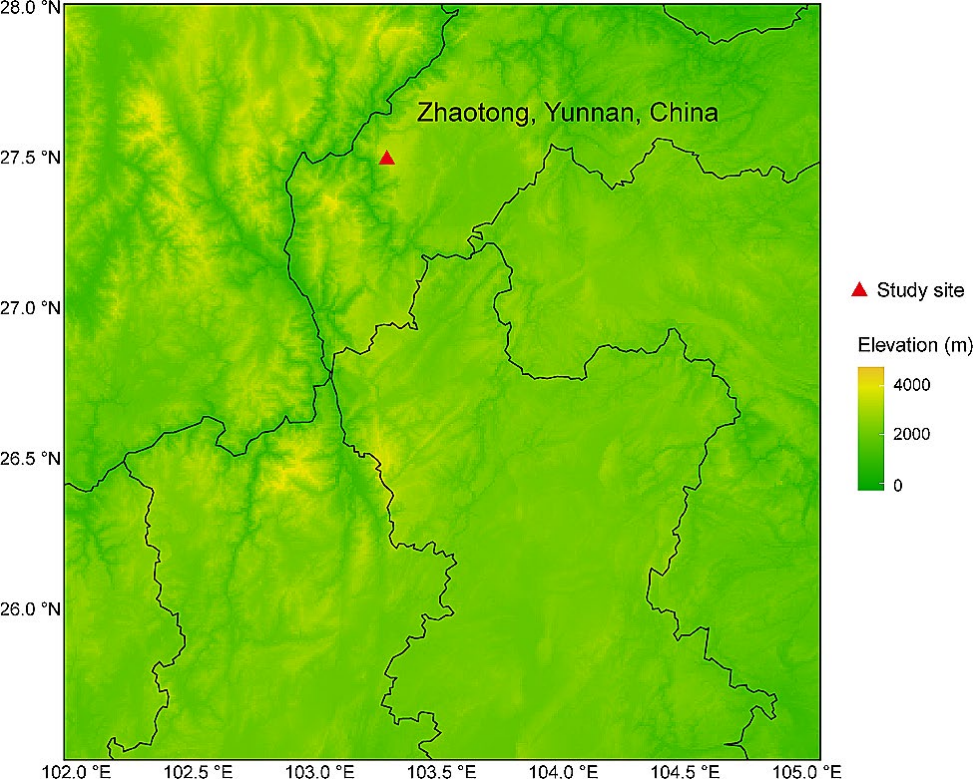
The sampling site for fresh feces of black-necked cranes

The Dashanbao Black-necked Crane National Nature Reserve (27° 18′ 38″–27° 28′ 42″ N, 103° 14′ 55″–103° 18′ 38″ E, altitudes of 3,000–3,200 m) is situated in the southwestern region of China (Fig. 1). In recent years, about 1,700 black-necked cranes have been wintering in the reserve every year [53]. In this study, fresh fecal samples of black-necked cranes were collected in Dashanbao Nature Reserve in Yunnan Province. A total of 40 samples were obtained during the wintering period of the black-necked cranes, with 10 samples collected in each month from December 2021 to March 2022. The collection of fecal samples was opportunistic; we continuously observed the black-necked cranes and collected samples using sterile gloves immediately after we found them defecating. To prevent duplicate sampling of the same individual, we implemented a sampling strategy with intervals greater than 5 m. Fresh fecal samples were collected and immediately placed into sterile bags at -20 °C. Within 2 h, the samples were transported to the laboratory and stored at -80 °C until DNA extraction. To prevent contamination from the external environment, the formal experiment focused on using the inner core of the fecal samples. According to the wintering process of black-necked cranes in Dashanbao, December is the early wintering period, January is the middle I wintering period, February is the middle II wintering period, and March is the late wintering period.

### DNA extraction and PCR amplification

Total fecal DNA extraction was performed using the QIAamp DNA stool minikit (Qiagen, Inc., Valencia, CA, USA) following the manufacturer’s guidelines. A negative control treatment was carried out with each extraction batch, containing all the same chemicals but without any DNA sample. Extracted DNA was quantifed and evaluated for purity using Qubit 2.0 Fluorometer (Life Technologies, Carlsbad, CA, USA) and gel electrophoresis, respectively. The trnL-P6 loop of plant was PCR-amplified using the following primers: trnL-c (5-GGAAGTAAAGTAAAAGTCGTAAAGG-3) and trnL-h (5-GCTGCGTTCTTCATCGATGC-3) [54]. The ITS1 region of fungi was PCR-amplified using the following primers: ITS5F (5-GGAAGTAAAGTAAAAGTCGTAAAGG-3) and ITS2R (5-GCTGCGTTCTTCATCGATGC-3) [55]. Both forward and reverse primers also contained a 5′ adaptor sequence to allow for subsequent indexing and Illumina sequencing. Each 20 µL PCR mixture contained 10 µL Phusion^®^ High-Fidelity PCR Master Mix (New England Biolabs), 1 µL of each forward and reverse primer, 6 µL of nuclease-free water, and 2 µL of DNA. PCR negative controls containing all reagents used in the PCR but no DNA were used to minimize the risk of contamination. DNA was PCR amplified using the following conditions for trnl sequences: initial denaturation at 94 °C for 3 min, followed by 40 cycles of 30 s at 94 °C, 30 s at 55 °C, and 1 min at 72 °C, and a final elongation at 72 °C for 10 min. DNA was PCR amplified using the following conditions for ITS1 sequences: initial denaturation at 98 °C for 1 min, followed by 40 cycles of 10 s at 98 °C, 30 s at 50 °C, and 30 s at 72 °C, and a final elongation at 72 °C for 5 min. The PCR products were pooled in equimolar concentrations on a 2% agarose gel, and purified PCR products were sequenced using the Illumina NovaSeq 6000 platform with paired end 2 × 250 bp reads (Illumina, United States) at Novogene (Beijing, China).

### Bioinformatics

The refdb package [56] is used for the construction of dietary reference databases. The datasets were download by using the following search terms in NCBI: txid33090 [ORGN] AND (trnL OR tRNA-Leu OR trn-L OR trn L) AND (chloroplast [Filter] OR plastid [Filter]) NOT environmental sample [Filter] NOT environmental samples [Filter] NOT environmental [Title] NOT uncultured [Title] NOT unclassified [Title] NOT unidentified [Title] NOT unverified [Title]. Then, the cleaning of the data was carried out to finally obtain the constructed dietary reference database. The barcode and primers of the raw data are removed using cutadapt [57]. Dada2 [58] is then employed for quality control, including removal of sequences containing Ns, sequences shorter than 100 bp for ITS sequences and 50 bp for trnl sequences, and low-quality sequences with “maxN = 0, maxEE = c(2, 2), truncQ = 2”. Subsequently, the paired-end sequences are merged and chimeras are removed to obtain ASVs (Amplicon Sequence Variants). The naive Bayesian classifier method was implemented for taxonomic assignment of ASVs based on the Unite database [59] for fungi data and plant database for diet data. Finally, to minimize the difference of sequencing depth across samples, the data was rarefied to the minimum read depths. Fungi were then rarefied to a sampling depth of 45,044 reads per sample and dietary data was rarefied to a sampling depth of 23,334 reads per sample for the downstream analysis.

### Statistical analysis

The vegan package [60] was utilized for alpha diversity analysis of plant diets and gut fungi, including the calculation of alpha diversity indices (Shannon, Simpson, Chao1, ACE) and the permanova analysis. The FUNGuild tool [61] was used to predict guild functions of fungal communities. Betadisper test was used to examine community dispersions of plant diets and gut fungi during different wintering periods in black-necked cranes. Kruskal-Wallis tests were conducted on different wintering periods, including alpha diversity and relative abundance of animal pathogens and wood saprotrophs. Subsequently, pairwise comparisons were performed using Dunn procedure. The Benjamini-Hochberg (bh) method was used for p-value correction in all multiple comparisons. Linear discriminant analysis (LDA) effect size (LEfSe) analysis was executed to assess differences at the genus level among different wintering periods [62]. Procrustes analysis and mantel test were employed to determine the correlation between diet and gut fungal. In addition, the Integrated Network Analysis Platform (INAP) [63] was employed to construct Molecular Ecological Network Analysis (MEAN) based on spearman correlation [64]. All fungi ASVs in the network were divided into four topological roles: peripherals (Zi < 2.5, Pi < 0.62), module hubs (Zi > 2.5, Pi < 0.62), connectors (Zi < 2.5, Pi > 0.62), and network hubs (Zi > 2.5, Pi > 0.62). Gephi [65] was used for the network visualization with the Fruchterman-Reingold layout. The visualization process was mainly realized using the “ggplot2” [66] in R [67].

## Results

### Diet diversity and composition of black-necked cranes during different wintering periods

**Fig. 2:**
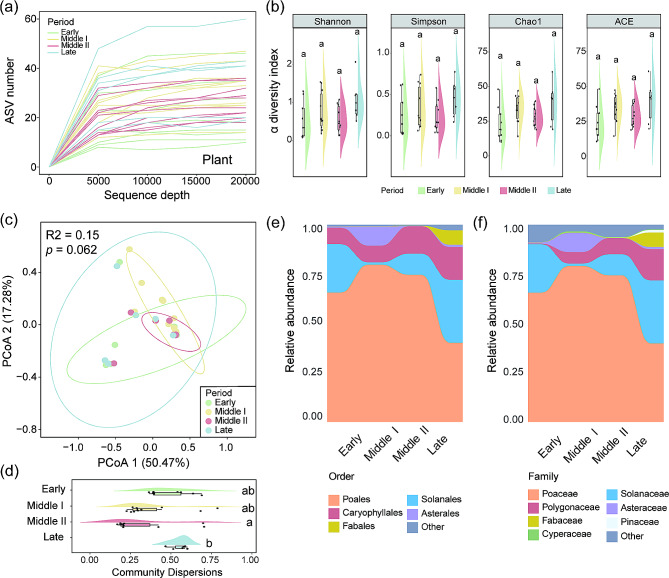
Dietary dynamics of black-necked cranes during the wintering period. (**a**) Rarefaction curve was used to evaluate the quality of sequencing depths. (**b**) Differences in dietary Shannon, Simpson, Chao1, and ACE indices among wintering periods. Values are presented as means ± sd. After a Kruskal-Wallis test, post hoc comparisons using Dunn test with a Benjamini-Hochberg correction indicated a significant difference among wintering periods, as indicated by the different letters (*p* < 0.05) (**c**) Principal coordinate analysis (PCoA) based on Bray-Curtis distances of plant Amplicon Sequence Variants (ASVs). (**d**) Differences in dietary community dispersions among wintering periods. The relative abundance of major plant (**e**) order and (**f**) family level taxa during wintering

Dietary information during the wintering period of black-necked cranes was recovered from 35 samples and a total of 3,212,326 (91,781 ± 4,256) raw reads were generated. After quality control and removal of chimeric sequences, a total of 2,858,314 (81,666 ± 14,443) clean reads were retained, accounting for 89.98% of the raw sequences. The results yielded a total of 378 (29 ± 12) ASVs, ranging from 10 to 60 ASVs per sample. Rarefaction curves of the number of plant ASVs with increasing sequence depth of samples, indicating that the rarefied sequence depth in our study capture most plant-based food from each sample (Fig. 2a). A total of 25 plant orders, 38 families, and 58 genera were identified from the 387 ASVs.

Alpha diversity analysis based on the Shannon, Simpson, Chao1, and ACE indices at the ASV level reveals no significant differences in the plant-based food of black-necked cranes during different wintering periods (Dunn test: *p* > 0.05; Fig. 2b and Table S1). Principal coordinate analysis (PCoA) based on Bray-Curtis distance at the ASV level revealed no distinct clustering pattern in the diet structure among different overwintering periods, and PERMANOVA analysis also indicated no significant differences (PERMANOVA: R2 = 0.15, *p* = 0.062; Fig. 2c). However, the diet community dispersions was significantly higher in the late wintering period compared to the middle II wintering period (Betadisper test: *p* < 0.05; Fig. 2d and Table S2).

**Fig. 3:**
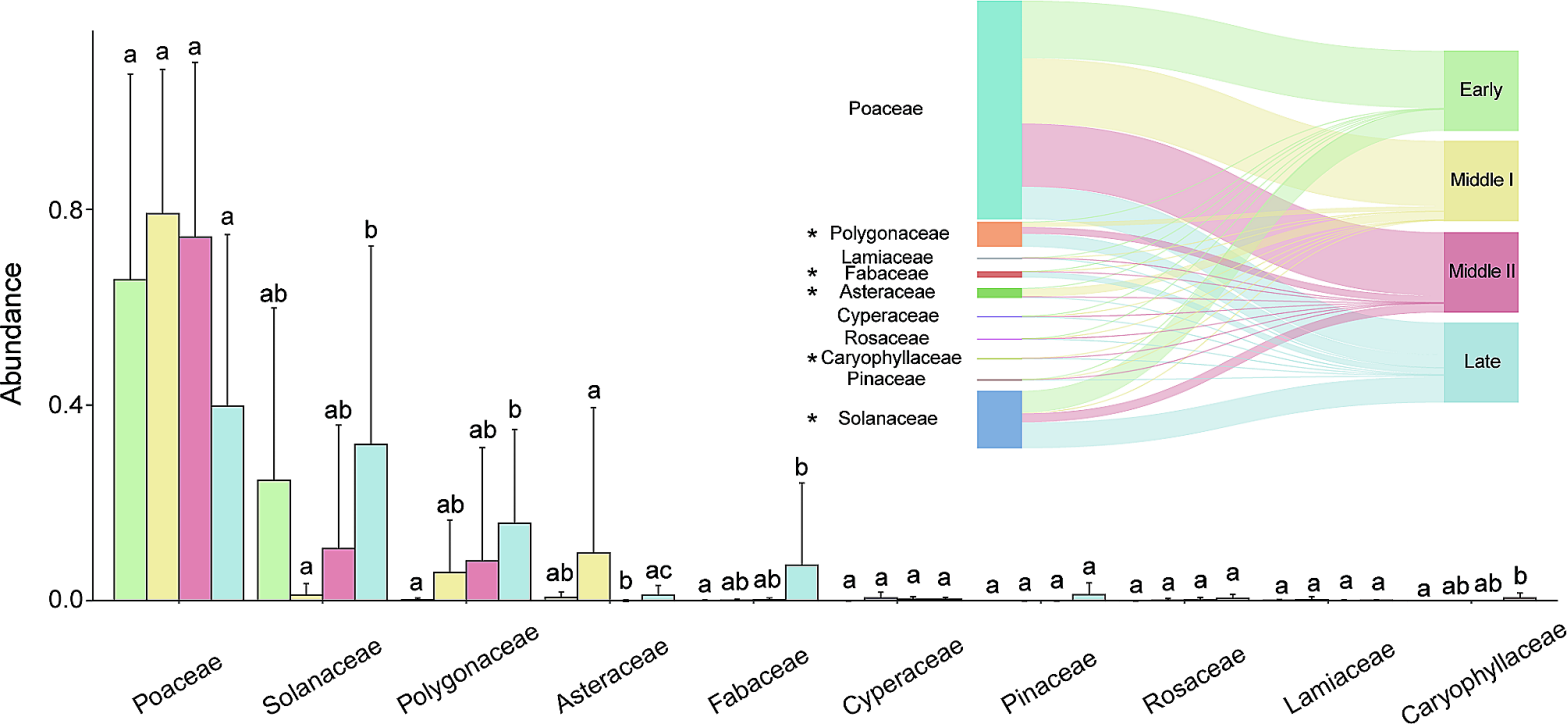
The differences in the top 10 plants in abundance during wintering at the family level

The major orders were Poales (relative abundance: 67.84% ± 36.87), Solanales (15.19% ± 28.99), and Caryophyllales (11.37% ± 18.33) (Fig. 2e). Black-necked cranes primarily consumed plants from the Poaceae (67.52% ± 36.80), Solanaceae (15.19% ± 28.99), and Polygonaceae (6.77% ± 15.90) (Fig. 2f). Among the top 10 most abundant plant families, significant differences were observed in the abundance of Solanaceae, Polygonaceae, Asteraceae, Fabaceae, and Caryophyllaceae among different wintering periods, with the highest abundance of Solanaceae, Polygonaceae, Fabaceae, and Caryophyllaceae occurring in the late wintering period (Fig. 3 and Table S3).

### Gut fungal diversity and composition of black-necked cranes during different wintering periods

**Fig. 4:**
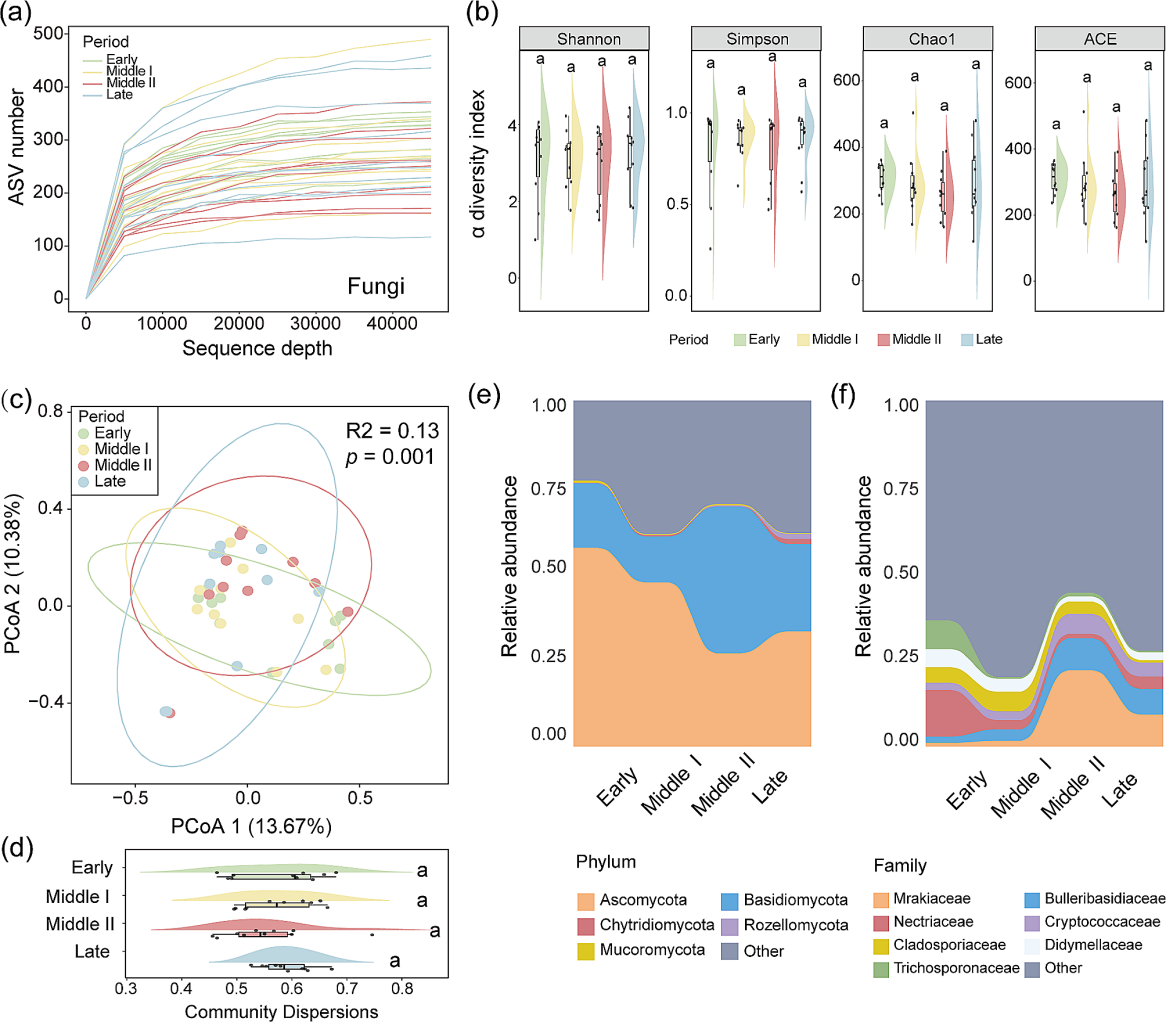
Fungal dynamics of black-necked cranes during the wintering period. (**a**) Rarefaction curve was used to evaluate the quality of sequencing depths. (**b**) Differences in fungal Shannon, Simpson, Chao1, and ACE indices among wintering periods. Values are presented as means ± sd. After a Kruskal-Wallis test, post hoc comparisons using Dunn test with a Benjamini-Hochberg correction indicated a significant difference among wintering periods, as indicated by the different letters (*p* < 0.05). (**c**) Principal coordinate analysis (PCoA) based on Bray-Curtis distances of fungal Amplicon Sequence Variants (ASVs). (**d**) Differences in fungal community dispersions among wintering periods. The relative abundance of major fungal (**e**) phylum and (**f**) family level taxa during wintering

Fungal data was obtained from 40 samples and a total of 3,784,614 (94,615 ± 4,327) raw reads were generated. After quality control and removal of chimeric sequences, a total of 2,903,788 (72,595 ± 11,329) clean reads were retained, accounting for 76.73% of the raw sequences. The results yielded a total of 4,970 (279 ± 80) ASVs, ranging from 117 to 490 ASVs per sample. Rarefaction curves of the number of fungal ASVs with increasing sequence depth of samples, indicating that the rarefied sequence depth in our study capture most fungi members from each sample (Fig. 4a). A total of 16 phyla, 51 orders, 120 orders, 274 families, 545 genera and 568 species were identified from 4,970 ASVs.

Alpha diversity analysis based on the Shannon, Simpson, Chao1, and ACE indices at the ASV level reveals no significant differences in the gut fungi of black-necked cranes during different wintering periods (Dunn test: *p* > 0.05; Fig. 4b and Table S4). PCoA analysis based on Bray-Curtis distance at the ASV level suggested no obvious clustering pattern in the community structure of gut fungi among different wintering periods, but PERMANOVA analysis showed significant differences (PERMANOVA: R2 = 0.13, *p* = 0.001; Fig. 4c). And there were no significant differences in gut fungi community dispersions among the four wintering periods (Dunn test: *p* > 0.05; Fig. 4d and Table S5).

The dominant gut fungal phyla were Ascomycota (Relative abundance: 41.25% ± 27.15) and Basidiomycota (24.91% ± 24.92) (Fig. 4e). The dominant families were Mrakiaceae (8.44% ± 16.73), Bulleribasidiaceae (5.44% ± 5.97), and Nectriaceae (5.25% ± 14.3) (Fig. 4f). The dominant genera were *Tausonia* (4.82% ± 15.57), *Fusarium* (4.73% ± 14.15), *Vishniacozyma* (4.12% ± 5.10), *Cryptococcus* (3.63% ± 4.23), and *Mrakia* (3.58% ± 6.46).

**Fig. 5:**
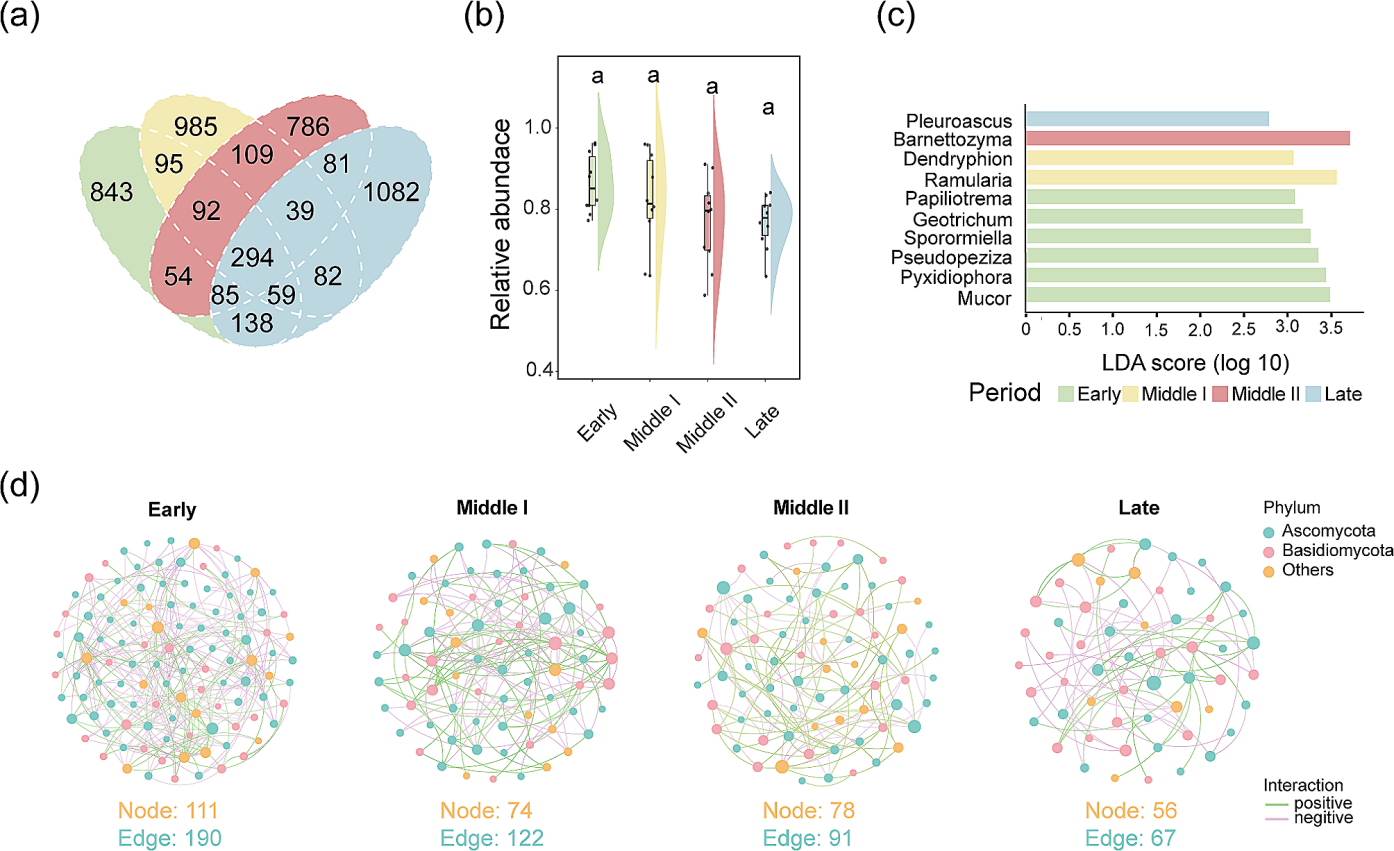
Shared core fungi (**a**) and their total abundance (**b**) of black-necked cranes during the wintering period. (**c**) Linear discriminant analysis (LDA) effect size (LEfSe) analyses of core fungi among wintering periods (LDA>2, *p*<0.05). (**d**) Molecular Ecological Network Analysis (MEAN) of gut fungi among different wintering periods

There were 294 fungal ASVs shared by black-necked cranes in different wintering periods (Fig. 5a), which accounted for a remarkably high proportion of the total abundance (early: 86.36% ± 7.30; middle I: 77.26% ± 10.33; middle II: 81.98% ± 11.15; late: 77.21% ± 6.34), and the difference in relative abundance among different wintering periods was not significant (Dunn test: *p* > 0.05; Fig. 5b and Table S6). We further performed a Lefse analysis on the core shared fungi at the genus level and identified 10 genera that differed significantly among wintering periods. Among them, *Mucor*, *Pyxidiophora*, *Pseudopeziza*, *Sporormiella*, *Geotrichum*, and *Papiliotrema* were significantly enriched in the early wintering period, *Ramularia* and *Dendryphion* were significantly enriched in the middle I wintering period, *Barnettozyma* was significantly enriched in the middle II wintering period, and *Pleuroascus* was significantly enriched in the late wintering period (Fig. 5c and Table S7).

The molecular ecological network of gut fungi in black-necked cranes during the wintering period was constructed based on the spearman correlation analysis of ASVs relative abundances. According to the number of nodes and edges, it was found that the network complexity was highest in the early and lowest in the late wintering period (Fig. 5d). Zi-pi analysis was used to identify key nodes in the molecular ecological networks of different wintering periods, revealing that seven ASVs were identified as connectors in the early wintering period, while only one connector was found in both middle II and late wintering periods (Fig. S2). These key taxa may play an important role in mediating the stability of the gut fungal molecular ecological network in black-necked cranes during wintering.

### Gut fungal pathogens of black-necked cranes during wintering

**Fig. 6:**
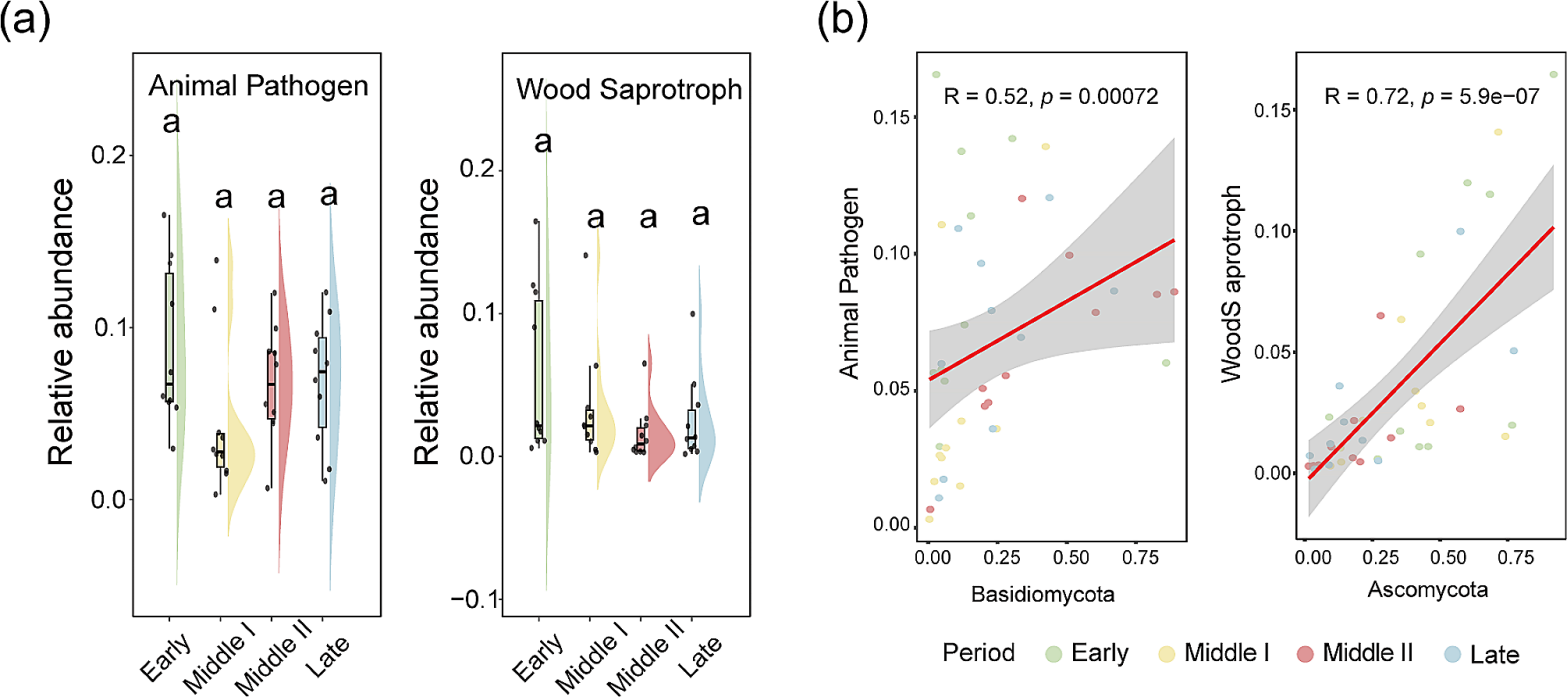
Dynamics of animal pathogen and wood saprotroph abundance (**a**) during wintering in the guild function. (**b**) The spearman correlation analysis between the abundance of animal pathogen and Basidiomycota and between the abundance of wood saprotroph and Ascomycota

The ecological functions of gut fungi in black-necked cranes during wintering were predicted using FunGuild. There were no significant differences in the relative abundance of animal pathogens and wood saprotrophs among different wintering periods, but their abundance was relative higher in the early wintering period (Dunn test: *p* > 0.05; Fig. 6a). Spearman correlation analysis revealed a positive correlation between the abundance of Basidiomycota and of animal pathogens (Spearman: *R* = 0.52, *p* = 0.00072), and between the abundance of Ascomycota and of wood saprotrophs (Spearman: *R* = 0.72, *p* = 5.9e − 07) (Fig. 6b).

### Relationship between diet and gut fungal composition of black-necked cranes during different wintering periods

**Fig. 7:**
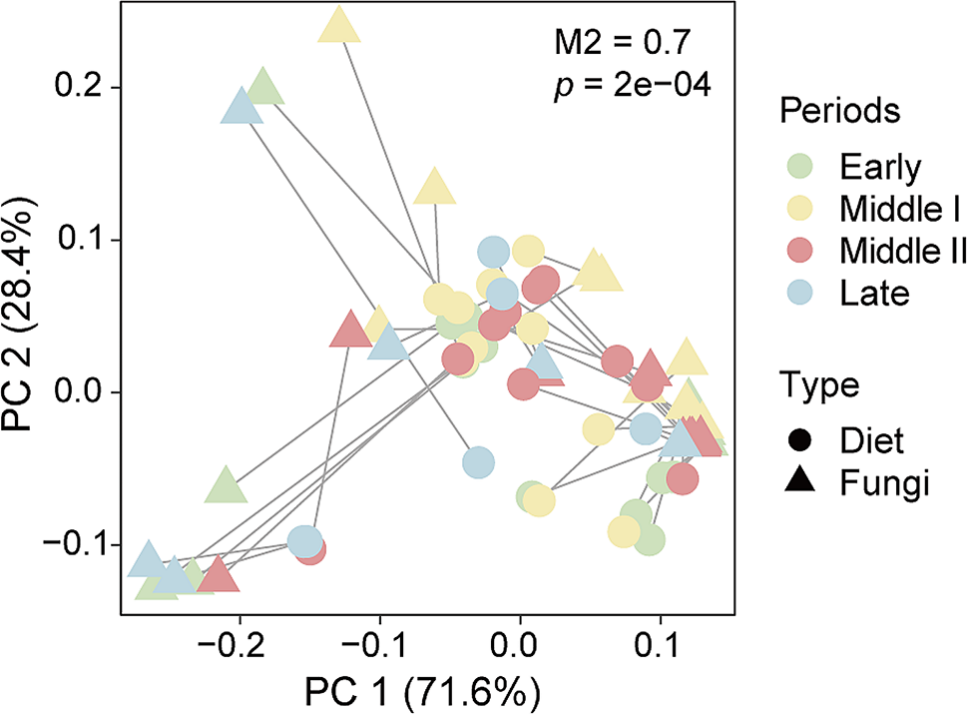
Procrustes analysis based on Bray-Curtis distances of the relative abundance of Amplicon Sequence Variants (ASVs) between diet and fungi

Procrustes analysis based on Bray-Curtis distance at the ASV level was performed to detect the correlation between diet and gut fungal communities during the wintering period of black-necked cranes, and the results showed that there was a significant correlation between them (Procrustes: M2 = 0.7, *p* = 2e − 04; Fig. 7). Mantel test also found a significant correlation between diet and fungi (Mantel: *R* = 0.503, *p* = 0.001). These suggest that the diet of black-necked cranes during the wintering period could play an active role in shaping the gut fungal community.

## Discussion

The changes in dietary composition have a significant impact on the intestinal microbial composition of birds [33, 68]. Black-necked cranes are omnivorous birds, but predominantly feed on plant-based foods [69]. Monitoring of animal feeding behavior can quantify the food consumed based on the time spent feeding, but with significant limitations in identifying the types of food consumed [31]. The DNA barcoding technique can further improve the identification of food types and quantification through relative percentages, offering higher accuracy and convenience compared to behavioral monitoring [18]. Therefore, in this study, we used high-throughput sequencing of the trnl gene in plant chloroplasts from fecal samples to systematically reveal the composition of plant-based food consumed by black-necked cranes during different wintering periods in Dashaanbao. The results showed that the plant-based food consumed by black-necked cranes during the wintering period mainly consisted of Poaceae, Solanaceae, Polygonaceae, Asteraceae, and Fabaceae, which is consistent with previous research [51]. We also found the abundance of major diets including Solanaceae, Polygonaceae, Fabaceae, and Caryophyllaceae increased significantly in the late wintering period, which represents a more balanced diet. Throughout the long process of evolution, animals have developed strategies to maximize energy intake from food, reduce or avoid high-fiber foods, and balance nutritional intake [69, 70]. In a study of the foraging strategies of black-necked cranes in Caohai, Guizhou, it was found that when natural food is scarce, black-necked cranes will forage in farmland [33]. In the Dashanbao area, we also found that black-necked cranes enter potato fields for foraging. As the wintering period progresses and the stock of potatoes in farmland gradually decreases, black-necked cranes adjust their foraging strategy by changing the types of food they consume. Observations of foraging behavior in Dashanbao black-necked cranes indeed showed a significant increase in the proportion of arthropod consumed and the time spent on this activity in the late wintering period [31]. These signals indicate that more pronounced changes in foraging strategy occur in the late wintering period. The decrease in preferred food in habitats and the increase in migratory pressure may lead black-necked cranes to broaden the variety of food intake in the late wintering period and increase their consumption of food they initially did not prefer. Furthermore, black-necked cranes show more pronounced individual dietary differences in the late wintering period, indicating that under environmental and migratory pressures in this period, they can ensure survival as much as possible through personalized dietary strategies.

In this study, we investigated the changes in the composition and structure of the gut fungal community of black-necked cranes during the wintering period in Dashanbao using the fungal ITS amplicon sequencing. The results showed that the diversity of gut fungal composition was relatively conserved during the wintering period of black-necked cranes, but the compositional structure differed significantly among wintering periods. Core fungi accounted for more than 75% in relative abundance among different wintering periods, while the proportion of unique fungi abundance in each period was relatively low. The taxonomic annotations of fungal ASVs with specificity for the wintering period identified the Parasola, some environmental saprophytic fungi (Thelephora and Acanthostigma), and lichen-parasitic fungi (Cyathicula) (Fig. S1) [71]. These fungal taxa were found only during one wintering period, which also suggests that the diversity of the fungal composition of animal gut detected from fecal samples may be influenced by endogenous fungi from food residues. The taxonomic annotation of the results showed that at the phylum level, Ascomycota, Basidiomycota, Chytridiomycota, Rozellomycota, and Mucoromycota were the dominant phyla, which is similar to previous research findings [10]. Differential abundance analysis revealed that the *Mucor*, *Pyxidiophora*, *Geotrichum*, and *Papiliotrema*, which are widely distributed in the soil, was significantly higher in the early wintering period, while *Pleuroascus*, which is widely distributed in arthropods, exhibited a significantly higher abundance in the late wintering period [71]. Monitoring feeding behavior also showed that black-necked cranes had a significantly higher proportion of potato consumption in the early period of overwintering, while consumption of arthropods was higher in the late wintering period [31]. These findings indicate that the gut fungal community structure of black-necked cranes is adapted to their feeding behavior. Consuming a large number of potatoes in the early period could promote the transfer of fungi widely distributed in the soil into the gut, resulting in a higher abundance. In the late wintering period, the higher consumption of arthropods could promote the transfer of fungi widely parasitizing in arthropods into the intestine, also resulting in a higher abundance [31]. The molecular ecological network of gut fungi was constructed, and it was found that the interrelationships among fungi gradually became simpler as winter progressed, and the analysis of the compositional structure of plant-based diet also showed that the proportion of consumption of various foods became more balanced in the late wintering period. These results suggest that a balanced food composition may play an essential role in stabilizing the community structure of gut fungi.

Gut fungal are closely associated with host immunity and dietary digestion [72–74]. In this study, we found that although the abundance of animal pathogens was highest in the early winter period compared to the other three periods, there were no significant differences between the wintering periods. Fungi from the phylum Ascomycota in the intestines have been shown to have high activity carbohydrase and decompose substances such as lignin and cellulose into unique small molecule metabolites in various animals, thereby helping the host improve food utilization [75, 76]. Meanwhile, through FunGuild for fungal functional prediction and Spearman correlation analysis, it was found that there is a significant positive correlation between Ascomycota and the richness of wood saprotrophs and animal pathogens, suggesting that Ascomycota fungi help hosts digest complex diets, thereby enhancing early winter adaptation to the environment while also potentially pathogenic, but further research is needed to reveal this.

Many studies have confirmed a strong correlation between bacterial communities in avian gut microbiota and host diet [77–79]. In this study, we also found a significant association between the diet composition of black-necked cranes and the composition of gut fungal. This suggests that food components may be a key factor influencing the gut fungal composition during the wintering period of black-necked cranes. For example, the phylum Ascomycota, which was widely abundant during all four wintering periods, can promote the decomposition of plant cellulose material, helping the host digest food and accumulate energy [43]. However, at the genus level, the detected dominant genus *Fusarium* is considered one of the world’s most pathogenic, phytotoxic, and toxigenic microbial groups [80]. The reports on its presence in animals are relatively lacking. *Vishniacozyma* and *Cryptococcus* have also been found in bird feces, but their specific mechanisms of action are still unclear [81]. More species of the genus *Mrakia* are found in extreme environments and food, while reports of them in animal intestines are relatively lacking [82]. Based on the above results, we also recognize the limitations of fecal examination in studying the fungal composition of wild animal intestines, as it can detect fungal taxa from food or soil ingested incidentally. It’s challenging to accurately differentiate fungi originating from incidental ingestion and those colonizing the intestine. This is also a limitation of this study, which could be addressed in future research by isolating and culturing relevant microbial groups for transplantation validation in animal models to assess their colonization in the intestine. Moreover, there are certain limitations in studying animal food composition based on DNA barcoding technology. Due to the relatively short molecular fragments, the identification efficiency at the species level is still quite limited [83, 84]. In the future, a comprehensive analysis can be conducted by combining macroscopic behavior monitoring and microscopic examination to further enhance the accuracy of food composition identification.

### Electronic supplementary material

Below is the link to the electronic supplementary material.

Supplementary Material 1

## Data Availability

The raw sequence data reported in this paper have been deposited in the Genome Sequence Archive (Genomics, Proteomics & Bioinformatics 2021) in National Genomics Data Center (Nucleic Acids Res 2022), China National Center for Bioinformation / Beijing Institute of Genomics, Chinese Academy of Sciences (GSA: CRA014379 and CRA014381) that are publicly accessible at https://ngdc.cncb.ac.cn/gsa.

## Abbreviations

LDA: Linear discriminant analysis
LefSe: Linear Discriminant Analysis (LDA) Effect Size
INAP: Integrated Network Analysis Platform
MEAN: Molecular Ecological Network Analysis
ASV: Amplicon sequence variants
PcoA: Principal coordinate analysis
ACE: Abundance-base Coverage Estimator
Chao 1: The Chao1 estimator

## Code availability

Code and detailed information are available on github (https://github.com/wenhaoli-wenhaoli/Gut-fungi-of-black-necked-cranes-Grus-nigricollis-respond-to-dietary-changes-during-wintering).
