# Supplementary material for: Gut fungi of black-necked cranes (Grus nigricollis) respond to dietary changes during wintering
Source: BMC Microbiol. 2024 Jun 29;24:232. doi: 10.1186/s12866-024-03396-0 (PMC11218170; doi:10.1186/s12866-024-03396-0)
Supplement: Supplementary file 1 — Supplementary Material 1 [file 12866_2024_3396_MOESM1_ESM.docx]

**Supplementary Material**. Gut fungi of black-necked cranes (*Grus nigricollis*) respond to dietary changes during wintering.

Wenhao Li^1, 3†^, Lijun Cheng^1, 2†^, Xin He^4^, Guiwen He^1, 2^, Zhenglin Sang ^1, 2^, Yuanjian Wang^5^, Mingcui Shao^5^, Tingsong Xiong^5^, Huailiang Xu^3*^, Junsong Zhao^1, 2*^

^1^ College of Agronomy and Life Sciences, Zhaotong University, Zhaotong 657000, China

^2^ Yunnan Key Laboratory of Gastrodia and Fungi Symbiotic Biology, Zhaotong University, Zhaotong 657000, China

^3^ College of Life Science, Sichuan Agricultural University, Ya’an 625014, China

^4^ Sichuan Academy of Grassland Sciences, Chengdu, 610000, China

^5^ Management Bureau of Dashanbao Black-Necked Crane National Nature Reserve, Yunnan Province, Zhaotong 657000, Yunnan, China

^†^These authors contributed equally to this work.

^*^Corresponding author:

Junsong Zhao, College of Agronomy and Life Sciences, Zhaotong University, Zhaotong 657000, China. Email: zhaojunsong@ztu.edu.cn

Huailiang Xu, College of Life Science, Sichuan Agricultural University, No. 46, Xinkang Road, Yucheng District, Ya’an, Sichuan 625014, China. Email: xuhuail@sicau.edu.cn


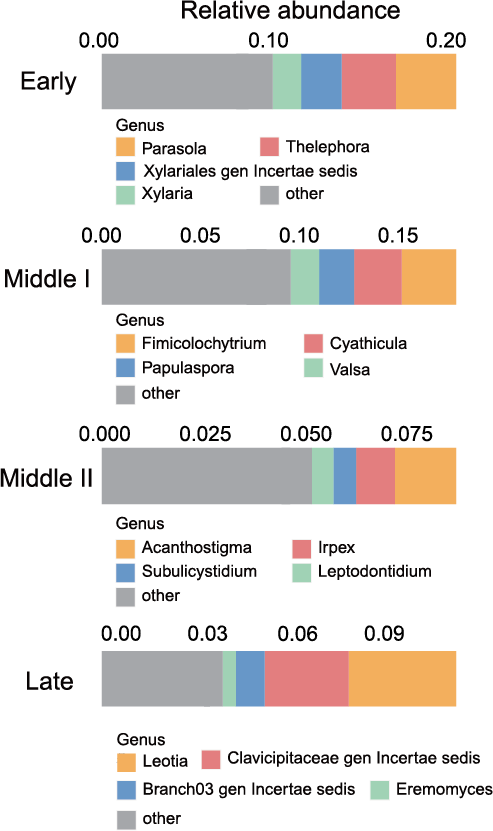


**Fig. S1** The abundance of specific fungal genera of the black-necked crane in each wintering period


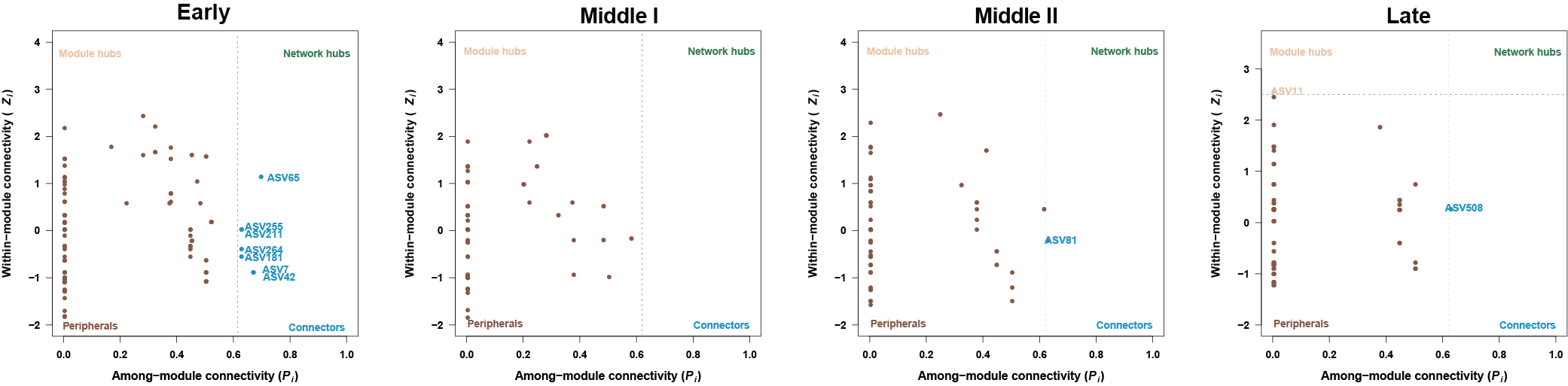


**Fig. S2** The key Amplicon Sequence Variants (ASVs) in fungal molecular ecological networks of black-necked cranes during each wintering period

**Table S1** Differences in dietary alpha diversity of black-necked cranes during the wintering period

| **α index** | **Comparison** | **Z** | **P.unadj** | **P.adj** |
| --- | --- | --- | --- | --- |
|  | Early - Middle II | -0.432 | 0.666 | 0.666 |
|  | Early - Middle I | -1.366 | 0.172 | 0.344 |
| Shannon | Middle II - Middle I | -0.960 | 0.337 | 0.505 |
|  | Early - Late | -2.068 | 0.039 | 0.232 |
|  | Middle II - Late | -1.726 | 0.084 | 0.253 |
|  | Middle I - Late | -0.895 | 0.371 | 0.445 |
|  | Early - Middle II | -0.163 | 0.871 | 0.871 |
|  | Early - Middle I | -1.289 | 0.198 | 0.395 |
| Simpson | Middle II - Middle I | -1.157 | 0.247 | 0.371 |
|  | Early - Late | -1.852 | 0.064 | 0.384 |
|  | Middle II - Late | -1.745 | 0.081 | 0.243 |
|  | Middle I - Late | -0.743 | 0.457 | 0.549 |
|  | Early - Middle II | -0.959 | 0.338 | 0.405 |
|  | Early - Middle I | -2.106 | 0.035 | 0.106 |
| Chao1 | Middle II - Middle I | -1.179 | 0.238 | 0.358 |
|  | Early - Late | -2.151 | 0.031 | 0.189 |
|  | Middle II - Late | -1.343 | 0.179 | 0.359 |
|  | Middle I - Late | -0.321 | 0.748 | 0.748 |
|  | Early - Middle II | -0.979 | 0.327 | 0.393 |
|  | Early - Middle I | -2.041 | 0.041 | 0.124 |
| ACE | Middle II - Middle I | -1.091 | 0.275 | 0.413 |
|  | Early - Late | -2.212 | 0.027 | 0.162 |
|  | Middle II - Late | -1.386 | 0.166 | 0.332 |
|  | Middle I - Late | -0.441 | 0.659 | 0.659 |

**Table S2** Differences in dietary community dispersions during wintering in black-necked cranes

| **Comparison** | **t** | **Observed p-value** | **permuted p-value** |
| --- | --- | --- | --- |
| Early - Middle II | 2.027 | 0.059 | 0.064 |
| Early - Middle I | 1.332 | 0.201 | 0.214 |
| Middle II - Middle I | 0.694 | 0.497 | 0.471 |
| Early - Late | -1.266 | 0.228 | 0.217 |
| Middle II - Late | -2.595 | 0.021 | 0.021 |
| Middle I - Late | -2.055 | 0.059 | 0.058 |

**Table S3** Differences in the main dietary families of black-necked cranes among wintering periods

| **Family** | **Comparison** | **Z** | **P.unadj** | **P.adj** |
| --- | --- | --- | --- | --- |
|  | Early - Middle II | 0.375 | 0.707 | 1.000 |
|  | Early - Middle I | 0.163 | 0.871 | 0.871 |
| Poaceae | Middle II - Middle I | -0.218 | 0.827 | 0.993 |
|  | Early - Late | 2.099 | 0.036 | 0.215 |
|  | Middle II - Late | 1.808 | 0.071 | 0.141 |
|  | Middle I - Late | 1.997 | 0.046 | 0.138 |
|  | Early - Middle II | -0.219 | 0.826 | 0.826 |
|  | Early - Middle I | 1.586 | 0.113 | 0.225 |
| Solanaceae | Middle II - Middle I | 1.855 | 0.064 | 0.191 |
|  | Early - Late | -1.265 | 0.206 | 0.309 |
|  | Middle II - Late | -1.096 | 0.273 | 0.328 |
|  | Middle I - Late | -2.703 | 0.007 | 0.041 |
|  | Early - Middle II | -1.767 | 0.077 | 0.116 |
|  | Early - Middle I | -1.575 | 0.115 | 0.138 |
| Polygonaceae | Middle II - Middle I | 0.196 | 0.844 | 0.844 |
|  | Early - Late | -3.426 | 0.001 | 0.004 |
|  | Middle II - Late | -1.925 | 0.054 | 0.109 |
|  | Middle I - Late | -2.095 | 0.036 | 0.109 |
|  | Early - Middle II | 2.389 | 0.017 | 0.051 |
|  | Early - Middle I | 0.197 | 0.844 | 0.844 |
| Asteraceae | Middle II - Middle I | -2.251 | 0.024 | 0.049 |
|  | Early - Late | -0.752 | 0.452 | 0.542 |
|  | Middle II - Late | -2.893 | 0.004 | 0.023 |
|  | Middle I - Late | -0.943 | 0.346 | 0.518 |
|  | Early - Middle II | -1.131 | 0.258 | 0.310 |
|  | Early - Middle I | -1.228 | 0.220 | 0.329 |
| Fabaceae | Middle II - Middle I | -0.099 | 0.921 | 0.921 |
|  | Early - Late | -2.707 | 0.007 | 0.041 |
|  | Middle II - Late | -1.757 | 0.079 | 0.237 |
|  | Middle I - Late | -1.670 | 0.095 | 0.190 |
|  | Early - Middle II | -2.021 | 0.043 | 0.086 |
|  | Early - Middle I | -2.216 | 0.027 | 0.080 |
| Cyperaceae | Middle II - Middle I | -0.200 | 0.842 | 0.842 |
|  | Early - Late | -2.258 | 0.024 | 0.144 |
|  | Middle II - Late | -0.506 | 0.613 | 0.920 |
|  | Middle I - Late | -0.333 | 0.739 | 0.887 |
|  | Early - Middle II | -0.445 | 0.656 | 0.984 |
|  | Early - Middle I | -0.181 | 0.856 | 0.856 |
| Pinaceae | Middle II - Middle I | 0.271 | 0.786 | 0.944 |
|  | Early - Late | -2.208 | 0.027 | 0.164 |
|  | Middle II - Late | -1.857 | 0.063 | 0.127 |
|  | Middle I - Late | -2.092 | 0.036 | 0.109 |
|  | Early - Middle II | -0.691 | 0.490 | 0.587 |
|  | Early - Middle I | 0.077 | 0.939 | 0.939 |
| Rosaceae | Middle II - Middle I | 0.789 | 0.430 | 0.646 |
|  | Early - Late | -2.105 | 0.035 | 0.106 |
|  | Middle II - Late | -1.533 | 0.125 | 0.250 |
|  | Middle I - Late | -2.216 | 0.027 | 0.160 |
|  | Early - Middle II | 0.597 | 0.551 | 0.826 |
|  | Early - Middle I | -0.440 | 0.660 | 0.660 |
| Lamiaceae | Middle II - Middle I | -1.065 | 0.287 | 0.861 |
|  | Early - Late | -0.967 | 0.333 | 0.667 |
|  | Middle II - Late | -1.518 | 0.129 | 0.774 |
|  | Middle I - Late | -0.595 | 0.552 | 0.662 |
|  | Early - Middle II | -1.848 | 0.065 | 0.194 |
|  | Early - Middle I | -1.752 | 0.080 | 0.120 |
| Caryophyllaceae | Middle II - Middle I | 0.099 | 0.921 | 0.921 |
|  | Early - Late | -3.312 | 0.001 | 0.006 |
|  | Middle II - Late | -1.736 | 0.083 | 0.099 |
|  | Middle I - Late | -1.822 | 0.068 | 0.137 |

**Table S4** Differences in fungi alpha diversity of black-necked cranes during the wintering period

| **α index** | **Comparison** | **Z** | **P.unadj** | **P.adj** |
| --- | --- | --- | --- | --- |
|  | Early - Middle II | 0.363 | 0.716 | 1.000 |
|  | Early - Middle I | 0.421 | 0.674 | 1.000 |
| Shannon | Middle II - Middle I | 0.057 | 0.954 | 0.954 |
|  | Early - Late | -0.172 | 0.863 | 1.000 |
|  | Middle II - Late | -0.536 | 0.592 | 1.000 |
|  | Middle I - Late | -0.593 | 0.553 | 1.000 |
|  | Early - Middle II | 0.459 | 0.646 | 1.000 |
|  | Early - Middle I | 0.861 | 0.389 | 1.000 |
| Simpson | Middle II - Middle I | 0.402 | 0.688 | 1.000 |
|  | Early - Late | 0.210 | 0.833 | 0.833 |
|  | Middle II - Late | -0.249 | 0.804 | 0.964 |
|  | Middle I - Late | -0.650 | 0.515 | 1.000 |
|  | Early - Middle II | 1.817 | 0.069 | 0.415 |
|  | Early - Middle I | 0.956 | 0.339 | 0.678 |
| Chao1 | Middle II - Middle I | 0.861 | 0.389 | 0.584 |
|  | Early - Late | 1.052 | 0.293 | 0.878 |
|  | Middle II - Late | 0.765 | 0.444 | 0.533 |
|  | Middle I - Late | 0.096 | 0.924 | 0.924 |
|  | Early - Middle II | 1.741 | 0.082 | 0.491 |
|  | Early - Middle I | 0.880 | 0.379 | 0.758 |
| ACE | Middle II - Middle I | -0.861 | 0.389 | 0.584 |
|  | Early - Late | 0.975 | 0.329 | 0.988 |
|  | Middle II - Late | -0.765 | 0.444 | 0.533 |
|  | Middle I - Late | 0.096 | 0.924 | 0.924 |

**Table S5** Differences in fungal community dispersions during wintering in black-necked cranes

| **Comparison** | **t** | **Observed p-value** | **permuted p-value** |
| --- | --- | --- | --- |
| Early - Middle II | 0.555 | 0.586 | 0.599 |
| Early - Middle I | -0.004 | 0.997 | 0.998 |
| Middle II - Middle I | 0.613 | 0.548 | 0.532 |
| Early - Late | -0.580 | 0.569 | 0.561 |
| Middle II - Late | -1.249 | 0.228 | 0.234 |
| Middle I - Late | -0.673 | 0.509 | 0.524 |

**Table S6** Differences in shared fungal abundance of black-necked cranes among wintering periods

| **Comparison** | **Z** | **P.unadj** | **P.adj** |
| --- | --- | --- | --- |
| Early - Middle II | 1.252 | 0.210 | 0.421 |
| Early - Middle I | 0.590 | 0.555 | 0.555 |
| Middle II - Middle I | 0.663 | 0.508 | 0.609 |
| Early - Late | 0.697 | 0.486 | 0.729 |
| Middle II - Late | 1.949 | 0.051 | 0.308 |
| Middle I - Late | 1.287 | 0.198 | 0.595 |

**Table S7** Fungal genera with significant differences in abundance during the wintering period

| **genus** | **period** | **LDA** | **p value** |
| --- | --- | --- | --- |
| *Papiliotrema* | Early | 3.080 | 0.042 |
| *Sporormiella* | Early | 3.259 | 0.007 |
| *Pseudopeziza* | Early | 3.350 | 0.008 |
| *Geotrichum* | Early | 3.170 | 0.004 |
| *Mucor* | Early | 3.484 | 0.033 |
| *Pyxidiophora* | Early | 3.435 | 0.036 |
| *Ramularia* | Middle I | 3.558 | 0.050 |
| *Dendryphion* | Middle I | 3.065 | 0.018 |
| *Barnettozyma* | Middle Ⅱ | 3.711 | 0.005 |
| *Pleuroascus* | Late | 2.786 | 0.019 |
